# Pathogen quantitative efficacy of different spike-in internal controls and clinical application in central nervous system infection with metagenomic sequencing

**DOI:** 10.1128/spectrum.01139-23

**Published:** 2023-11-20

**Authors:** Zhangfan Fu, Jingwen Ai, Haocheng Zhang, Peng Cui, Tao Xu, Yumeng Zhang, Yi Zhang, Honglong Wu, Ao Shen, Ke Lin, Miaoqu Zhang, Chao Qiu, Ning Jiang, Yang Zhou, Wenhong Zhang

**Affiliations:** 1 Department of Infectious Diseases, National Medical Center for Infectious Diseases, Shanghai Key Laboratory of Infectious Diseases and Biosafety Emergency Response, Huashan Hospital, Shanghai Medical College, Fudan University, Shanghai, China; 2 BGI PathoGenesis Pharmaceutical Technology, BGI-Shenzhen, Shenzhen, China; 3 National Clinical Research Center for Aging and Medicine, Huashan Hospital, Fudan University, Shanghai, China; 4 Key Laboratory of Medical Molecular Virology (MOE/MOH), Shanghai Medical College, Fudan University, Shanghai, China; 5 Shanghai Huashen Institute of Microbes and Infections, Shanghai, China; Beijing Institute of Genomics, Chinese Academy of Sciences, Beijing, China

**Keywords:** mNGS, spike-in, internal control, quantitation, central nervous system infection

## Abstract

Metagenomic next-generation sequencing (mNGS) has been a lack of method for pathogen quantitation. We explored the suitable concentration of *T_1_ phage* [internal control (IC)_phage_], *Thermus thermophilus* (IC_T.T_), and artificial DNA sequence (IC_DNA_) as mNGS ICs for pathogen quantitation and compared the quantitation efficiency among them. We prepared the simulated cerebrospinal fluid (CSF) samples composed of a pathogen cocktail, containing *Staphylococcus aureus*, *Escherichia coli*, *Komagataella pastoris*, and human cells to test the accuracy, linearity, and interference of IC quantitation. We also collected 15 clinical CSF conducting both mNGS and droplet digital PCR (ddPCR) to further verify the quantification efficacy of IC. In accuracy, the mNGS quantification of pathogen was more precise when the IC was 10^3^ and 10^4^ CFU/mL *Thermus thermophilus* or 10^3^ and 10^4^ PFU/mL *T_1_ phage* with the CV% of pathogens quantification most below 15%. The DNA sequence’ quantification was less accurate with all the CV% of pathogen quantitation above 15%. In linearity, compared to DNA sequence (all *R* ^2^ <0.9), 10^3^ CFU/mL *Thermus thermophilus* and 10^4^ PFU/mL *T_1_ phage* both have stronger linearity in mNGS pathogen quantification according to linear regression (most *R* ^2^ >0.9). In interference, the mNGS quantification was affected obviously by human cell concentrations when the IC was DNA sequence (*P* < 0.0001), and the quantification was not interfered when the IC was the 10^3^ CFU/mL *Thermus thermophilus* or 10^4^ PFU/mL *T_1_ phage*. Furthermore, we revealed the mNGS quantitation was highly consistent with ddPCR in clinical CSF according to the linear regression (*R* ^2^ = 0.9646, *P* < 0.0001, *k* = 0.9362) and Bland-Altman (the bias of average difference is 4.033, with the 95% confidence interval from − 24.61 to 32.68). *Thermus thermophilus* and *T_1_ phages* are comparable as mNGS IC in pathogen quantitation and are both superior than artificial DNA sequences. In total, 1,000-CFU/mL *Thermus thermophilus* as mNGS IC may allow us to reflect directly the variation of pathogens in central nervous system infection patients.

**IMPORTANCE:**

Metagenomic next-generation sequencing (mNGS) has been used broadly for pathogens detection of infectious diseases. However, there is a lack of method for the absolute quantitation of pathogens by mNGS. We compared the quantitative efficiency of three mNGS internal controls (ICs) *Thermus thermophilus*, *T1* phages, and artificial DNA sequence and developed the most applicable strategies for pathogen quantitation via mNGS in central nervous system infection. The IC application strategy we developed will enable mNGS analysis to assess the pathogen load simultaneously with the detection of pathogens, which should provide critical information for quick decision-making of treatment as well as clinical prognosis.

## INTRODUCTION

Quantitative detection of pathogens provides critical data for microbial burden assessment in clinical specimens, facilitating estimation of the severity and progress of infectious disease. Conventional methods for quantitation of microorganisms rely on microbial culture, microscopy counting, quantitative polymerase chain reaction (qPCR), droplet digital PCR (ddPCR), etc., which usually depend on the diagnosis of pathogens in advance or have limited targets of detection.

Metagenomic next-generation sequencing (mNGS) has been widely used in the detection of pathogens in various infectious diseases, including bloodstream infection (1), focal infection (2), central nervous infection (3, 4), and respiratory infection (5, 6), and facilitated quick and accurate infection diagnosis. mNGS pathogen analysis provides quantitative and semi-quantitative data via the counting of sequenced reads or the relative abundance of a targeted pathogen in polymicrobial samples. Reports have demonstrated the mNGS semi-quantitation of pathogens in cerebrospinal fluid (CSF) (4), thereby monitoring disease progression. However, unbiased metagenomic quantitation could be impacted by factors including pathogens’ genome sizes and human cell count variation between specimen types (7).

In order to achieve accurate pathogen detection and quantitation by mNGS across different samples, researchers have developed various spike-ins as internal controls (ICs). Several types of spike-ins have been mainly involved in mNGS analysis, including artificial DNA sequences, DNA sequences of 23 bacterial and three archaeal species (MBARC-26) (8), bacteria and viruses that are rarely found in clinical environment [such as *Thermus thermophilus HB8* genome DNA (9) and *T_1_ phages* and *MS_2_ phages* combined (10)], and high range RNA ladder consisting of λ phages and plasmids (11). While the DNA sequence spike-ins were mostly used for quality control purposes of mNGS library construction and sequencing, there are few efficiency assessments of various ICs in pathogen quantitation in clinical specimens. Information regarding the types, concertation, and timepoint of the addition of mNGS quantitation ICs is required for clinical practice, as well.

A desirable mNGS quantitative IC should be easily accessible, stable to detect, have low homology to the human and pathogenic organisms, and have few impacts on the specificity and sensitivity of detection. In this study, we chose *T_1_ phage*, *Thermus thermophilus*, and artificial DNA sequence as candidate ICs, all of which are rarely or never detected in clinical samples. *T_1_ phage* and *Thermus thermophilus* can be easily cultured and quantified under laboratory conditions, which is significantly different from the human body environment. Artificial DNA sequences are widely applied mNGS ICs due to their low homology to known genomes. We explored the relatively suitable concentration of *T_1_ phage*, *Thermus thermophilus*, and artificial DNA sequence as mNGS ICs and compared the pathogen quantitation efficiency among these three spike-ins. The spike-in with the best quantitation performance in simulated samples was further tested in clinical CSF samples to verify its quantitation accuracy.

## MATERIALS AND METHODS

### Strains and culture preparation

The pathogen cocktail including *Staphylococcus aureus* (ATCC 29213, *S. aureus*), *Escherichia coli* (ATCC 11303, *E. coli*), and *Komagataella pastoris* (ATCC 28485, *K. pastoris*) was purchased from American Typical Culture Collection (ATCC). *S. aureus* and *E. coli* were cultured in lysogeny broth (LB) media for 6 hours at 37°C at 200 rpm, and *K. pastoris* (ATCC 28485) was cultured in LB media at 30°C. The concentrations of spiking bacteria and fungi were measured by counting of colony forming units (CFU) on agar LB plates after 24-hour or 48-hour culture at 37°C.

The internal controls used in the study included the *T_1_ phage* (ATCC 11303-B1), *Thermus thermophilus* (CGMCC 1.6492), and artificial DNA sequence, all of which were spiked separately into the CSF simulated samples and clinical CSF specimens before the DNA extraction. *T_1_ phage* was bought from ATCC. *Thermus thermophilus* was bought from the China General Microbiological Culture Collection Center (CGMCC). The artificial DNA sequence was bought from BGI. The *T_1_ phage* was cultured in *E. coli* (optical density [OD] value ranging from 0.1 to 0.3) at 37°C at 200 rpm for 6 hours, and the *Thermus thermophilus* was cultured in No. 697 media recommended by ATCC at 70°C for 48 hours.

The cells used as the composition of the human genome material in the simulated sample were the residual lymphocytes separated from healthy peripheral blood obtained from clinical laboratory from healthy volunteers in Fudan University Affiliated Huashan Hospital.

### The count method of microbial cells of stimulated samples

We cultured *S. aureus*, *K. pastoris*, and *E. coli* in 5 mL of LB media until they reached the late logarithmic growth phase. Then, 3 mL of bacteria was diluted to 30 mL PBS with a gradient of 10 times and plated 200 µL of the diluted samples onto three separate LB agar media plates for each dilution. We incubated the plates at 37°C overnight, except for the *K. pastoris* plates, which required at least 48 hours of incubation. We preserved each dilution by storing them immediately in a −80°C refrigerator. Finally, we counted the number (*N*) of CFU on each plate and used the formula *C* = *N*_mean_ × 5 × dilution times to calculate the concentration (CFU/mL) of each microorganism.

### The elimination of phage host gene

To obtain a bacterial cell-free phage lysate, the first step involves centrifuging the cultured T1 phage solution at 4,000 g for 5 minutes. Afterward, the supernatant is filter-sterilized using a 0.22-mm filter, specifically the Whatman Anotop 0.02-mm sterile syringe filters (cat. no. 09-926-13; Fisher Scientific). Next, the phage solution is needed to perform ultrafiltration, and 15 mL of the phage solution is added to the upper reservoir of the Amicon filter device (cat. no. UFC910008; Millipore). The Amicon is then centrifuged at 4,000 g for approximately 5 minutes. The resulting filtrate is carefully discarded into a waste bucket, and SM buffer is added until the volume reaches 15 mL. This wash step is repeated three times, and the phage solution is concentrated to 200 µL. Finally, to eliminate any host genes present, DNAase (18047019, Invitrogen) is used according to the provided instructions and allowed to digest for 30 minutes at 37℃.

### Clinical samples

All 15 clinical CSFs were collected from patients at Fudan University Affiliated Huashan Hospital with the diagnosis of suspected central nervous system (CNS) infection from 7 March 2017 to 1 August 2018 after getting the consent of the patients and surrogates. All patients were enrolled in clinical trial NCT03232242, and all samples were residual specimens, with the presence of at least one bacterial or fungal pathogen according to the previous mNGS results. The volumes of all samples were sufficient to extract DNA for both mNGS and ddPCR tests.

### Simulated samples of central nervous system infection

The artificial CSF simulated samples were prepared with a pathogen cocktail and human cells. The microbial cells were added to the simulated CSF in a 10-fold dilution series (Tabels S1 to S3).

#### Library preparation and sequencing

Simulated samples and clinical CSF specimens were stored at −80°C, and 600 µL simulated or clinical samples were removed into a sterile 2.0 mL centrifuge tube and mixed with 1 g 0.5 mm BioSpec beads (0.5 mm dia. zirconia/silica, cat. no. 11079105z) before being agitated vigorously at 2,800–3,200 rpm for 30 minutes on a horizontal platform on a Vortex-Genie 2 Vortex Mixer 12 (Scientific Industries, NY, USA). TIANamp Micro DNA Kit (DP316, Tiangen Biotech, Beijing, China) was used to extract total DNA.

DNA libraries were generated by DNA fragmentation, end repair, A-tailing addition, adapter ligation, and PCR amplification. Quality control was undertaken by the Agilent 2100 and Qubit 2.0 system, and qualified libraries (200–300 bp, >2 ng/mL) were sequenced on the BGISEQ-200 platform with a single-end 50 bp strategy. Each sample yielded at least 20 million reads. To control the sequencing quality and contamination of each sequencing run, we added positive and negative control (HeLa cell lines with or without *Acinetobacter baumannii*) in each run.

#### Bioinformation pipeline

High-quality sequencing data were obtained by filtering low-quality and short (length <35 bp) reads. Following quality processing, by using the Burrows-Wheeler alignment (Version 0.7.17), the clean reads were mapped to human reference databases including hg19 and Yanhuang genome sequence, and the remaining reads were aligned to the non-redundant bacterial, viral, fungal, and parasite databases. The mapping data were processed in preparation for advanced data analysis. The genome databases were downloaded from the National Center for Biotechnology Information (NCBI) . RefSeq contains 4,945 whole genome sequences of viral taxa, 6,350 bacterial genomes or scaffolds, 1,064 fungi related to human infection, and 235 parasites associated with human diseases. The raw data were uploaded to China National GeneBank (CNP0000607, CNP0000610).

#### Criteria for a positive mNGS results

The sequencing results from each sample were organized into two tables, one for bacteria/fungi and the other for viruses. The specific mapped read number (SMRN) for each microbial taxonomy was standardized to SMRN per 20 million (M) of total sequencing reads, creating the standardized SMRN (SDSMRN), calculated using the formula: SDSMRN = SMRN × 20 million/total reads.

To be considered as positively detected in bacterial/fungal analysis, the microbe had to meet four criteria: (i) be among the top 10 genera with the highest SDSMRN, (ii) be ranked first within its genus, (iii) have an SDSMRN >1, and (iv) be a commonly reported infection pathogen.

For virus analysis, a virus was considered positively detected if it met two criteria: (i) belonged to the top 3 with the highest SDSMRN and (ii) had an SDSMRN >5.

Despite the low yield of DNA extraction and the risk of contamination, pathogens like *Mycobacterium* spp., *Nocardia* spp., *Brucella* spp., and others were still identified if they met three criteria: (i) were among the top 20 genera with the highest SDSMRN, (ii) were ranked first within their respective genus, and (iii) had an SDSMRN >1. For the detection of pathogens within the *Enterobacteriaceae* family, only the species with the highest SDSMRN was considered a positive detection.

### Evaluation of microbial quantitation performance of mNGS using ICs

#### The precision of microbial quantitation at different concentrations

The simulated samples (10^4^ cells/mL of human cells, 10^2^ CFU/mL of pathogen cocktail containing equivalent *S. aureus*, *E. coli*, and *K. pastoris*) were divided into three groups spiked with one of the three quantitation ICs, the *T_1_ phage* (IC_Phage_), *Thermus thermophilus* (IC_T.T_), or artificial DNA sequence (IC_DNA_). The IC_phage_ was at concentrations of 10^3^, 10^4^, 10^5^, and 10^6^ PFU/mL; the IC_T.T_ was at concentrations of 10^2^, 10^3^, and 10^4^ CFU/mL; and the IC_DNA_ was at concentrations of 1.7, 6.7, and 24.7 ng/mL (Table S1). We made six replicates mNGS test of each concentration of IC. The mNGS quantitation results were compared with the known concertation of microbial cells in the simulated samples. The precision of quantitation was defined with a percent coefficient of variation (% CV) of no more than 15%. The best concentration for each IC was then chosen for the linearity and human concentration tests.

#### The linearity of mNGS quantitation

The CSF simulated samples contained 10^4^ cells/mL of human cells and pathogen cocktails (equivalent concentration of *S. aureus*, *E. coli*, and *K. pastoris*) at the concertation of 10^2^, 10^3^, or 10^4^ CFU/mL. One of the three quantitative ICs, the *T_1_ phage* (10^4^ PFU/mL), *Thermus thermophilus* (10^3^ CFU/mL), or artificial DNA sequence (6.7 ng/mL), was spiked into each set of simulated CSFs (Table S2). The linear regression models were generated to access the linearity of quantitation of mNGS by a coefficient of determination (*R* ^2^).

#### Impact of human cells on mNGS quantitation

CSF-simulated samples with different human cell concentrations (10^3^, 10^4^, 10^5^, or 10^6^ cells/mL) were used to testify to the influence of the human genome background on the quantitation efficiency of mNGS. Pathogen cocktail (equivalent concentration of *S. aureus*, *E. coli*, and *K. pastoris*) at 10^2^ CFU/mL was added to all CSF simulated samples. Three ICs (10^4^ PFU/m *T_1_ phage* l, 10^3^ CFU/mL *Thermus thermophilus*, and 6.7 ng/mL artificial DNA sequence) were spiked into each set of samples (Table S3). Analysis of variance (ANOVA) test was used to evaluate the effect of human cell concentration of samples on the mNGS’ absolute quantitation.

### Droplet Digital PCR (ddPCR)

ddPCR assays were performed on the D3200 ddPCR platform [Pilot Gene Technologies (Hangzhou) Co., Ltd.] to measure the copy numbers of *S. aureus*, *E. coli*, and *Cryptococcus neoformans* (*C. neoformans*) in clinical CSF samples. ddPCR nucleic acid detection kit [Pilot Gene Technologies (Hangzhou) Co., Ltd.] was used according to the manufacturer’s instructions.

### Statistical analysis

In this study, we calculated the pathogen concentration of mNGS tests by the following formula:


$$C_{M}=\left(C_{IC}\times L_{IC}\times N_{M}\right)/\left(N_{IC}\times L_{M}\right)$$

*C*_*M*_ is the concentration of the pathogen, *C*_IC_ is the concentration of IC, *L*_*M*_ is the average genome length of the calculated pathogen, *L*_IC_ is the genome length of IC, *N*_*M*_ is the read number of the calculated pathogen, and *N*_IC_ is the read number of IC.

The difference between measured and theoretical concentration of pathogens equals the absolute value of the value, which is the theoretical concentration cut measured value of pathogen concentration.

Statistical analysis was performed with GraphPad Prism 8.0 and Excel 2021. Quantitative agreement and linearity between ddPCR and mNGS were estimated using linear regression and Bland-Altman plots.

## RESULTS

### The precision of mNGS pathogen quantitation

In this part, we intended to find the most appropriate concertation of ICs that could precisely quantify the pathogen load. The simulated samples with the same concentration of human and pathogen cells were divided into three groups, each spiked with *T_1_ phage*, *Thermus thermophilus*, or artificial DNA sequence (Table S1). Six replicated mNGS tests were conducted with each concentration of ICs.

Taking *T. thermophilus* as IC (IC_T.T_), as the concentration of IC_T.T_ was 10^2^, 10^3^, and 10^4^ CFU/mL, the CV% of *S. aureus* quantitation by mNGS was 9.81%, 5.58%, and 6.72%, that of *K. pastoris* quantitation was 12.94%, 10.24%, and 21.69%, and that of *E. coli* quantitation was 11.70%, 12.86%, and 4.56%, respectively (Table 1). When IC_T.T_ was at 10^3^ CFU/mL, the CV% of *S. aureus* and *K. pastoris* quantitation was the lowest, indicating the most accurate quantitation of these two pathogens. Nevertheless, the most accurate concentration of IC_T.T_ for *E. coli* quantitation was 10^4^ CFU/mL, but this concentration resulted in a CV% above 15% for *K. pastoris*, thus indicating inaccurate quantitation of the pathogen.

**TABLE 1:** The CV% value of gradients IC_T.T_’s quantification

Concentration of IC	10^2^ CFU/mL	10^3^ CFU/mL	10^4^ CFU/mL
*S. aureus*	9.81%	5.58%	6.72%
*E. coli*	11.70%	12.86%	4.56%
*K. pastoris*	12.94%	10.24%	21.69%

Secondly, we use *T_1_ phage* as quantitative IC of mNGS (IC_Phage_), and four concentrations of the IC_Phage_ (10^3^, 10^4^, 10^5^, and 10^6^ PFU/mL) were chosen for precision analysis. With the increasing concentration of IC_Phage_, the CV% of mNGS quantitation was 3.09%, 6.78%, 9.29%, and 2.94% for *S. aureus*, respectively. The CV% was 4.47%, 12.4%, 16.32%, and 15.28% for *K. pastoris* and was 6.75%, 5.25%, 14.91%, and 21.79% for *E. coli* (Table 2). Overall, the quantitation was more accurate at 10^3^ PFU/mL and 10^4^ PFU/mL of IC_phage_. However, when the IC_phage_ increased to 10^5^ PFU/mL and 10^6^ PFU/mL, the CV% of *E. coli* and *K. pastoris* quantitation were close to or above 15%, and therefore the quantitation was not accurate.

**TABLE 2:** The CV% value of gradients IC_phage_’s quantification

Concentration of IC	10^3^ PFU/mL	10^4^ PFU/mL	10^5^ PFU/mL	10^6^ PFU/mL
*S. aureus*	3.09%	6.78%	9.29%	2.94%
*E. coli*	6.75%	5.25%	14.91%	21.79%
*K. pastoris*	4.47%	12.40%	16.32%	15.28%

At last, artificial DNA sequence was utilized as mNGS IC (IC_DNA_) to quantitate pathogens. The concentration of IC_DNA_ was 1.7, 6.7, and 26.7 ng/mL. The CV% of *S. aureus*, *E. coli*, and *K. pastoris* quantification (ranging from 21.11% to 52.99%, Table 3) was higher than 15% with all three concentrations of IC_DNA_, which means the quantification was not accurate. We suspected that the inaccuracy of IC_DNA_ might be due to the lack of complete bacteria or virus structure, which induced different efficiencies of nucleic acid extraction from pathogens. Based on these results, we proposed that bacteria or virus structure is a crucial feature for mNGS ICs in precise pathogen quantitation.

**TABLE 3:** The CV% value of gradients IC_DNA_’s quantification

Concentration of IC	1.7 ng/mL	6.7 ng/mL	26.7 ng/mL
*S. aureus*	21.60%	45.86%	36.46%
*E. coli*	25.76%	34.96%	21.11%
*K. pastoris*	22.27%	52.99%	47.08%

In conclusion, we discovered that 10^2^ CFU/mL, 10^3^ CFU/mL of IC_T.T_, and 10^3^ PFU/mL and 10^4^ PFU/mL of IC_T phage_ provide better precision of pathogen quantitation than IC_DNA_. Considering the quantitative accuracy and mNGS limit of detection, we selected 10^3^ CFU/mL *Thermus thermophilus* and 10^4^ PFU/mL *T_1_ phage* to continue the quantitative linearity investigation and selected 6.7-ng/mL DNA artificial sequence additionally as the control IC for the subsequent study.

### The linearity of mNGS quantitation

After the confirmation of the IC concertation with the acceptable quantitation precision, we tried to test if the mNGS provides accurate quantitation by measuring the linearity of a measured value to theoretical pathogen concentration.

**Fig 1:**
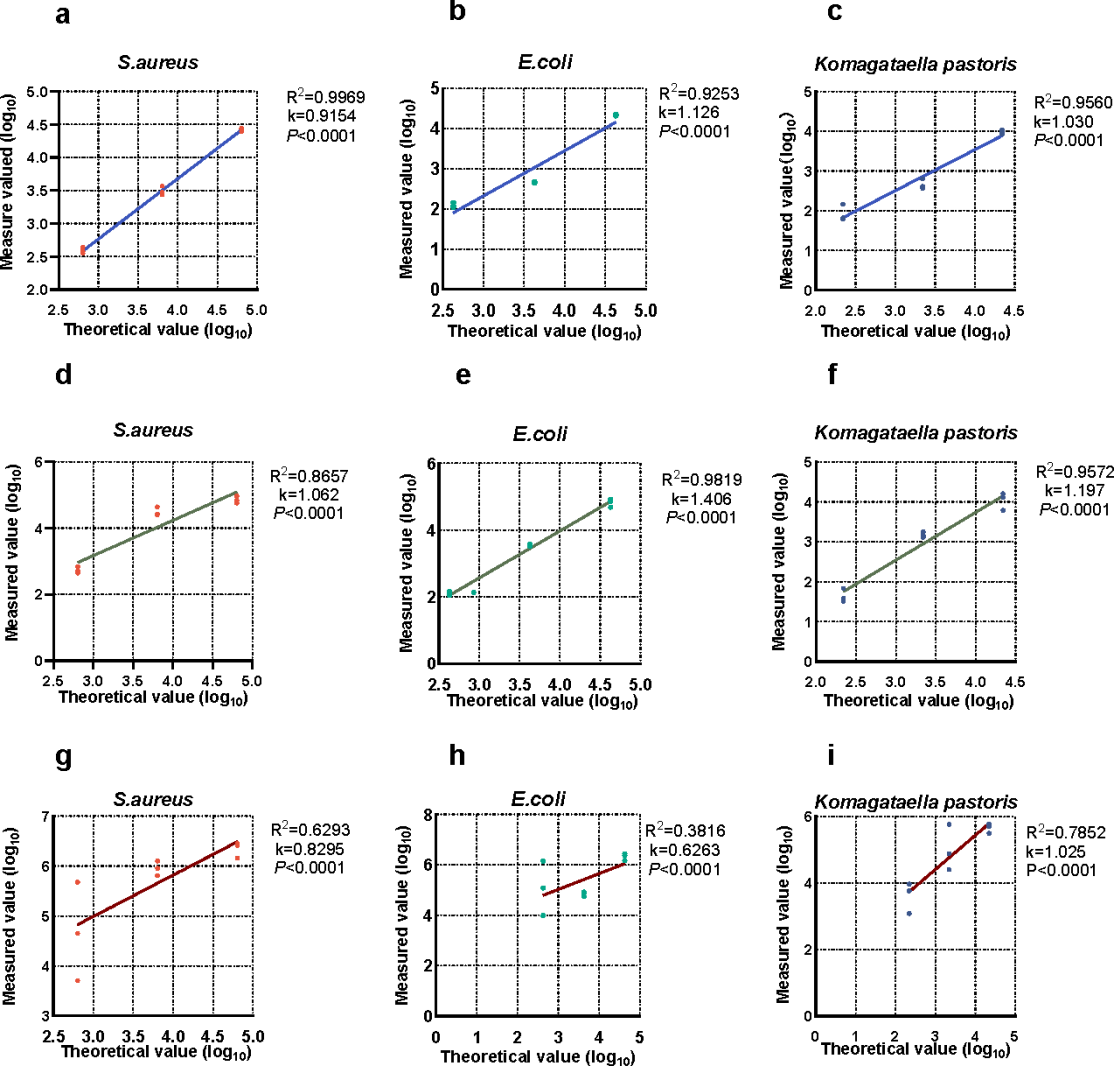
The linearity of mNGS quantitation with three ICs. The red, green, and blue dot plots showed the measured and theoretical values of *S. aureus*, *E. coli*, and *K. pastoris* quantitation. The linearity of mNGS quantitation with 10^3^ CFU/mL IC_T.T_ (**a, b, c**), 10^4^ PFU/mL IC_phage_ (**d, e, f**), and 6.7 ng/mL IC_DNA_ (**g, h, i**) as ICs was accessed by linear regression presented by the blue solid, green, and red lines, respectively.

When IC_T.T_ (10^3^ PFU/mL) was used, strong linearity of mNGS quantitation of *S. aureus* (*R* ^2^ = 0.9969, *k* = 0.9154, *P* < 0.0001), *E. coli* (*R* ^2^ = 0.9253, *k* = 1.126, *P* < 0.0001), and *K. pastoris* (*R* ^2^ = 0.9560, *k* = 1.030, *P* < 0.0001) was observed with their theoretical concentration (Fig. 1a through c). mNGS quantitation with IC_phage_(10^4^ CFU/mL) was also good in linearity when quantifying *S. aureus*, *E. coli*, and *K. pastoris*: (*R* ^2^ = 0.8657, *k* = 1.062, *P* < 0.0001; *R* ^2^ = 0.9819, *k* = 1.406, *P* < 0.0001; *R* ^2^ = 0.9572, *k* = 1.197, *P* < 0.0001; Fig. 1d through f). Although significant linearity was also observed with artificial DNA sequence (*P* < 0.0001), the *R* ^2^ were all below 0.9 (*R* ^2^ = 0.6189, 0.6263, and 0.7852; Fig. 1g through i) when quantifying the pathogen cocktail.

In general, 10^3^ CFU/mL IC_T.T_ and 10^4^ PFU/mL IC_phage_ were more accurate and stable in mNGS quantitation, compared to the artificial DNA sequence.

### Impact of human cells on mNGS quantitation

The human genome had a significant influence on the sensitivity of pathogen detection via unbiassed metagenomics and may impact the quantitation efficiency of ICs. In this part, we evaluated the impacts of the human cell concentrations on mNGS quantitation with a series of simulated CSF (human cell concentrations were 10^3^, 10^4^, 10^5^, and 10^6^ cells/mL) containing 10^2^ CFU/mL of pathogen cocktail. In addition, 10^3^ CFU/mL *Thermus thermophilus*, 10^4^ PFU/mL *T_1_ phage*, and 6.7 ng/mL artificial DNA sequence were added as mNGS ICs.

**Fig 2:**
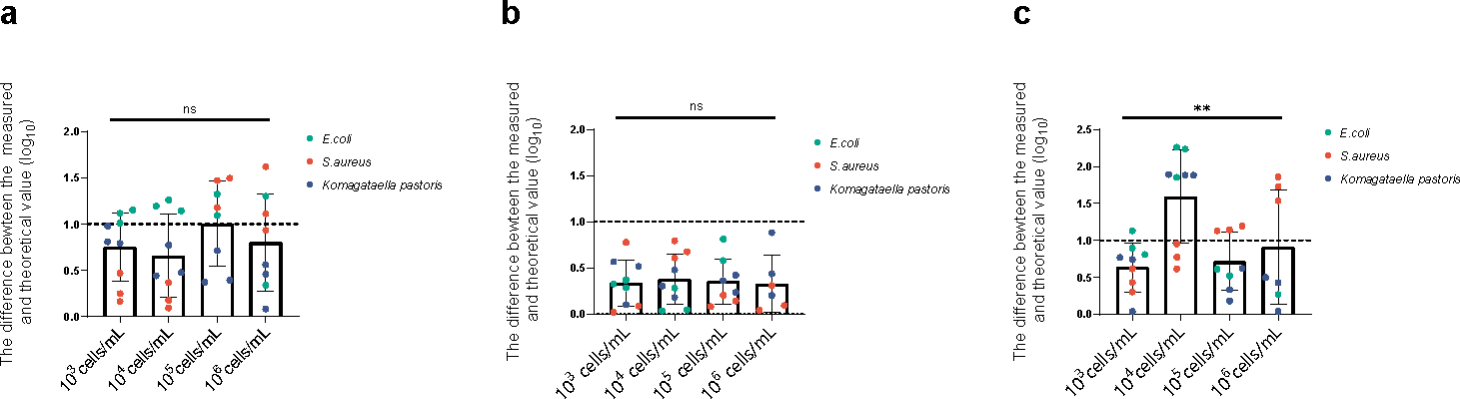
The impact of human cell concentrations on mNGS quantification. The red, green, and blue dot plots showed that the difference between the mNGS measured and the theoretical value of *S. aureus*, *E. coli*, and *K. pastoris*. In addition, 10^3^ CFU/mL IC_T.T_ (**a**), 10^4^ PFU/mL IC_phage_ (**b**), and 6.7 ng/mL IC_DNA_ (**c**) were used as mNGS IC. And these different results were analyzed by ANOVA. The ns and ** present no significance and obvious significance, respectively. The dashed line expresses the limitation of quantification accuracy by mNGS.

The detected reads number of ICs and pathogens were significantly impacted by the human genome background. With the increase of human cell concentration, the detected read of ICs and pathogens decreased (Fig. S2). But overall, the impact of human genome concentrations (ranging from 10^3^ to 10^6^ cells/mL）on the quantitative efficiency was minor. The difference between the mNGS measured value and theoretical concentration of pathogens was calculated and compared across human cell concentrations via the ANOVA analysis (Fig. 2). When IC_T.T_ at 10^3^ CFU/mL or IC_phage_ at 10^4^ CFU/mL was used for quantitation, the differences between measured and theoretical concentration of pathogen were not significantly impact by human genome amount (Fig. 2a and b). However, mNGS pathogen quantitation with IC_DNA_ was impacted by the human genome background (*P* < 0.0001), with the largest difference between the measured and theoretical concentration at 10^4^ /mL of human cells (Fig. 2c).

### Performance of mNGS quantitation in clinical specimens

#### Basic characteristics of clinical CSF specimens

A total of 15 CSF specimens with positive pathogenic infections were enrolled in this trial and classified into three groups, namely, the *S. aureus-*, *E. coli-,* and *Cryptococcus neoformans*-positive group, based on the prior mNGS results, in the Huashan Hospital of Shanghai from February 2021 to January 2022 (Table 4). All the baseline characteristics and diseases of the three groups were shown in Table 5, and there was not any significant difference between the groups including gender, age, white blood cell (WBC) count, C-reactive protein, erythrocyte sedimentation rate, and procalcitonin (Table 5).

**TABLE 4:** Final diagnosis of 15 patients with suspected central nervous system infections

Disease	Case numbers
Viral meningitis	1
Disseminated cryptococcal infection	1
Tuberculous meningitis with bacterial meningitis	1
Bacterial brain abscess	1
Bacterial meningitis	1
Cryptococcal meningitis	7
Bloodstream infection	1
Central nervous system vasculitis	1
Autoimmune encephalitis	1

**TABLE 5:** The baseline of suspected CNS infection CSF

	Total average (SD)	*E. coli* average (SD)	*S. aureus* average (SD)	*C. neoformans* average (SD)	*P* value
Person number	15	3	4	8	
WBC (10^6^ /L)	63.00 (96.53)	10.33 (13.80)	51.00 (56.65)	97.33 (131.02)	0.463
The ratio of mononuclear cells (%)	18.51 (36.00)	0.46 (0.65)	0.41 (0.36)	48.65 (48.54)	0.195
Glucose (mmol/L)	2.68 (0.95)	2.63 (0.38)	3.45 (0.70)	2.20 (1.04)	0.122
Cl (mmol/L)	116.38 (10.27)	117.00 (5.29)	110.50 (13.99)	120.00 (8.99)	0.388
Protein (mg/L)	1979.23 (2350.93)	1159.33 (851.64)	3462.25 (3923.33)	1400.50 (1083.58)	0.342

#### The quantitative agreement and linearity between ddPCR and mNGS

**Fig 3:**
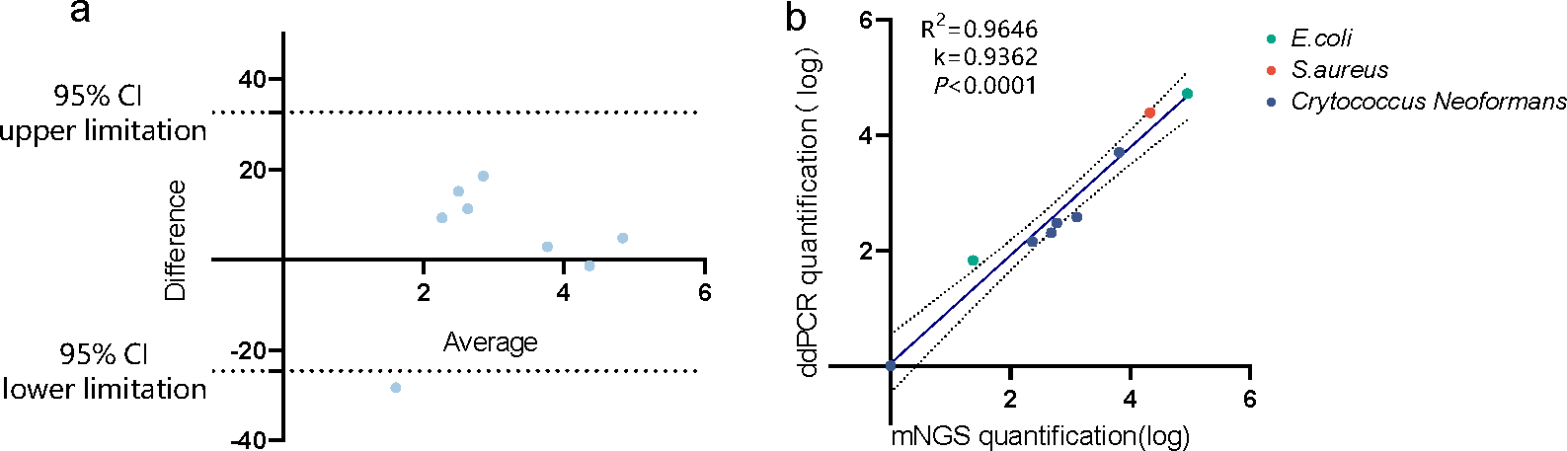
The quantitation accuracy of mNGS with 10^3^ CFU/mL IC_T.T_ in clinical CSF. The agreement between mNGS quantitation and ddPCR quantification analyzed by Altman-Bland (a) demonstrated the bias of average difference of 4.033 of both methods (all dots), with the 95% limits of agreement (dotted line) between the methods ranging from −24.61 to 32.68. The linear regression (b) showed a regression line of *y* = 0. 9362× *x+* 0.04225 , with a slope from 0.7758 to 1.097, with an intercept ranging from -0.4646 to 0.5491. The solid line and dotted line displayed the regression line and 95% confidence intervals, respectively. The red, green, and blue dots represented the DNA copies number of *S. aureus*, *E. coli*, and *C.neoformans*, respectively.

Comprehensively considering the quantitative performance of all the ICs, 10^3^ CFU/mL *IC_T.T_* and 10^4^ CFU/mL IC_phage_ exhibited good precision and linearity and were unaffected by human genome concentration. However, the IC*_phage_* solution was prone to contamination of host *E. coli* DNA (Fig. S1). Hence, the mNGS quantitation was conducted with 10^3^ CFU/mL IC_T.T_ in CSF clinical samples. The amount of pathogen was simultaneously measured by ddPCR. The mNGS quantitation of *S. aureus*, *E. coli*, and *C. neoformans* was highly consistent with ddPCR by the Bland-Altman test, which showed that most points of the test were within 95% confidence interval (Fig. 3a). The quantitation of mNGS also kept excellent linearity with ddPCR on quantifying different concentrations of pathogens in CSF samples. According to the linear regression analysis of all 15 specimens, the measured value of ddPCR and mNGS had good linearity (*R* ^2^ = 0.9646, *P* < 0.0001, *k* = 0.9362; Fig. 3b). These results also manifested that the absolute quantitation of mNGS had an agreement with ddPCR in quantifying different pathogens.

## DISCUSSION

Quantitative detection of pathogens in the clinical specimens could provide critical information for etiology diagnosis, disease severity assessment, and progression monitoring. Although clinical mNGS analysis could provide sequence numbers of detected pathogens, the impacts of human genome background, the varied features of pathogen cells, and differences in sampling operation challenged its application in quantitative pathogen detection, especially absolute quantitation. Several research groups tried to develop mNGS quantitation techniques for clinical pathogen detection. A recent study on urine samples was conducted to complete a semi-quantitative pathogen detection by mNGS via receiver operating characteristic curve analyses, in which a diagnostic index was calculated to determine what a cutoff value of reads was in urine culture positive. Meanwhile, a few researchers focused on developing appropriate quantitative spike-in for mNGS (12). For example, the internal reference External RNA Controls Consortium (ERCC) can identify the suspected pathogens by quantifying each component of contaminating microorganisms (13). What’s more, some Chinese researchers developed a spike-in internal control, which was positively correlated with input DNA quantity but inversely correlated with the amount of host cells (7). The quantitation of transplant-related viral DNA by Galileo mNGS pipeline, which comprises IC, external control, and software that enables virus identification was reported before (14).

A desirable mNGS quantitative IC should have several features: (i) the nucleic acid in the IC being stable to detect; (ii) nucleic acid sequences with low homology to the genome of human and pathogenic organisms; and (iii) little negative impacts on the sensitivity of detection. This study evaluated the mNGS quantitation with three ICs, *T_1_ phage*, *Thermus thermophilus*, and artificial DNA sequence, in simulated CSF, and further testified the mNGS quantitation with 10^3^ CFU/mL IC_T.T_ on clinical specimens for achieving pathogen absolute quantitation. The artificial DNA sequence was stable and low in sequence homology. However, the precision of mNGS quantitation with artificial DNA sequence was poor and more impacted by human genome concentration. These limitations of artificial DNA sequence might be due to its relatively short sequence length and lack of cell structures, which render its read number unpredictable in sequencing. In fact, quite a few mNGS pathogen detection pipeline uses artificial DNA for quality control purposes of library construction and sequencing, instead of pathogen quantitation. *T_1_ phage* is another quantitative IC we adopted, which had good precision, linearity, and stability of pathogen detection. However, although the sequence of *T_1_ phage* genome was rarely detected in tissue and humoral specimens from humans, the *T_1_ phage* suspension inevitably contains some nucleic acid sequences from the host *E. coli*. These *E. coli* sequences might result in false-positive detection of pathogenic *E. coli* (Fig. S1)*. Thermus thermophilus* was a rare environmental bacterial species mainly habituating in the hot springs. *Thermus thermophilus* has low homology to most human pathogens but a whole cellular structure, and our results confirmed that mNGS quantitation with IC_T.T_ had good pathogen quantitation efficiency. And quantitation of bacteria and fungi by mNGS can be accomplished using sequence reads normalized by 10^3^ CFU/mL IC_T.T_.

There are a few limitations in the study. To begin, the study exclusively focuses on CNS infection patients due to the relatively low nucleic acids of CSF and absence of backgroud microbes. Next, the validation cohort just enrolled 15 mNGS positive cases, which is insufficient to avoid accidental errors in verifying the quantitation capability of mNGS. Third, the classification of pathogens of the validation cohort only included the *S. aureus*, *E. coli*, and *C. neoformans* mNGS positive, despite the fact that they stand for the Gram-positive, Gram-negative, and yeast fungi, respectively. Finally, we did not further explore the precision of absolute quantitation in the lower limit of detection by (LLod) of mNGS, when the samples were spiked with ICs. Based on the limitation mentioned above, we should increase the types of specimens, such as bronchoalveolar lavage fluid (BALF), urine, blood, and spume, and broaden the validation cohort by increasing the sample number. Moreover, we should encompass more pathogens in the validation cohort especially the *Aspergillus* spp., which has a biological structure that is far different. We also can further explore the precision of quantitation in LLod of mNGS.

In summary, IC_T.T_ and IC_phage_ were comparable in pathogen quantitation and were both superior to IC_DNA_; nevertheless, IC_phage_ might introduce *E. coli* contamination into clinical specimens. Using the 1,000 CFU/mL IC_T.T_ as mNGS quantitative IC may allow us to directly reflect the variation of pathogens in CNS infection patients.

## Data Availability

The original contributions presented in the study are included in the article/Supplementary Material. Further inquiries can be directed to the corresponding author.

## SUPPLEMENTAL MATERIAL

Supplemental file 1 spectrum.01139-23-s0001.docx Supplemental methods, Tables S1 to S3, and Fig. S1 and S2

ASM does not own the copyrights to Supplemental Material that may be linked to, or accessed through, an article. The authors have granted ASM a non-exclusive, world-wide license to publish the Supplemental Material files. Please contact the corresponding author directly for reuse.
