# Supplementary material for: Pathogen quantitative efficacy of different spike-in internal controls and clinical application in central nervous system infection with metagenomic sequencing
Source: Microbiol Spectr. 2023 Nov 20;11(6):e01139-23. doi: 10.1128/spectrum.01139-23 (PMC10714923; doi:10.1128/spectrum.01139-23)

**Supplementary methods**

**Strains and Culture Preparation**

The pathogen cocktail including *Staphylococcus aureus* (ATCC 29213, *S. aureus*), *Escherichia coli* (ATCC 11303, *E. coli*) and *Komagataella pastoris* (ATCC 28485, *K. pastoris*) which were all purchased from American Typical Culture Collection (ATCC).  *S. aureus*, *E. coli* were cultured in LB media for 6 hours at 37℃ at 200rpm and *K. pastoris* (ATCC 28485) was cultured in LB media at 30℃. The concentrations of spiking bacteria and fungi were measured by counting of colony forming units(CFU) on agar LB plates after 24h or 48h culture at 37℃.

The internal controls used in the study included the *T_1_ phage* (ATCC 11303-B1), *Thermus thermophilus* (CGMCC 1.6492) and artificial DNA sequence, all of which were spiked separately into the CSF simulated samples and clinical CSF specimens before the DNA extraction. *T_1_ phage* was bought from ATCC. *Thermus thermophilus* was bought from China General Microbiological Culture Collection Center (CGMCC). artificial DNA sequence was bought from BGI. The *T_1_ phage* was cultured in *E.coli* (OD value ranging 0.1 to 0.3) at 37℃ at 200 rpm for 6 hours and the *Thermus thermophilus* was culture in No.697 media recommended by ATCC at 70℃ for 48 hours.

The cells used as the composition of the human genome material in the simulated sample were the residual lymphocytes obtained from clinical laboratory from patients diagnosed with non-infectious diseases in Fudan University Affiliated Huashan Hospital.

**Clinical samples**

All 15 clinical CSFs were collected from patients at Fudan University Affiliated Huashan Hospital with the diagnosis of suspected central nervous system (CNS) infectionfrom Mar 7, 2017, to Aug 1, 2018 after getting the consent of the patients and surrogates. All patients were all enrolled from clinical trial NCT03232242 and all samples were residual specimens, with the presence of at least one bacterial or fungal pathogen according to the previous mmNGS results. The volumes of all samples were sufficient to extract DNA for both mNGS and ddPCR tests.

**Library preparation and sequencing**

Simulated samples and clinical CSF specimens were stored at −80°C. 600 uL simulated or clinical samples were removed into a sterile 2.0 ml centrifuge tube and mixed with 1 g 0.5 mm BioSpec beads (0.5mm dia. zirconia/silica, Cat. No. 11079105z) before being agitated vigorously at 2,800–3,200 rpm for 30 min on a horizontal platform on a Vortex-Genie 2 Vortex Mixer 12 (Scientific Industries, USA). TIANamp Micro DNA Kit (DP316, Tiangen Biotech, Beijing, China) was used to extract total DNA.

DNA libraries were generated by DNA fragmentation, end repair, A-tailing addition, adapter ligation, and PCR amplification. Quality control was undertaken by the Agilent 2100 and Qubit 2.0 system, and qualified libraries (200–300 bp, >2 ng/mL) were sequenced on BGISEQ-200 platform with single-end 50 bp strategy. Each sample yielded at least 20 million reads. To control the sequencing quality and contamination of each sequencing run, we added positive and negative control (HeLa cell lines with or without *Acinetobacter baumannii*) in each run.

**Bioinformation Pipeline**

High-quality sequencing data were obtained by filtering low-quality and short (length <35 bp) reads. Following quality processing, by using the Burrows–Wheeler alignment (Version 0.7.17), the clean reads were mapped to human reference databases including hg19 and Yanhuang genome sequence and remaining reads were aligned to the non-redundant bacterial, viral, fungal, and parasite databases. The mapping data were processed in preparation for advanced data analysis. The genome databases were downloaded from National Center for Biotechnology Information (NCBI) (<ftp://ftp.ncbi>.nlm.nih.gov/genomes/). RefSeq contains 4945 whole genome sequence of viral taxa, 6350 bacterial genomes or scaffolds, 1064 fungi related to human infection, and 235 parasites associated with human diseases. The raw data was uploaded to China National GeneBank (CNP0000607).

**Statistical analysis**

In this study, we calculated pathogen concentration of mNGS tests by the following formula:

C_M_=${{{（C}_{IC}\times L_{IC}\times N_{M}）}/{{（N}_{IC}\times L}}_{M}）$

C_M_：the concentration of pathogen；C_IC_：concentration of IC；L_M_：the average genome length of calculated pathogen；L_IC_：the genome length of IC；N_M_: reads number of calculated pathogen; N_IC_：reads number of IC

Statistical analysis was performed with GraphPad Prism 8.0 and Excel 2021. Quantitative agreement and linearity between ddPCR and mNGS were estimating using linear regression and Bland-Altman plots.

Supplementary Table 1 the CSF simulated samples for exploring the most suitable concentration of spike-ins.

| Spike-ins | The concentration of spike-ins | *S.* *aureus* (CFU/mL) | *E. coli* (CFU/mL) | *K. pastoris* (CFU/mL) | Human cells (/mL) |
| --- | --- | --- | --- | --- | --- |
| *Thermus thermophilus* | 10^2^ CFU/mL | 10^2^ | 10^2^ | 10^2^ | 10^4^ |
| *Thermus thermophilus* | 10^3^ CFU/mL | 10^2^ | 10^2^ | 10^2^ | 10^4^ |
| *Thermus thermophilus* | 10^4^ CFU/mL | 10^2^ | 10^2^ | 10^2^ | 10^4^ |
| *T_1_ phage* | 10^3^ PFU/mL | 10^2^ | 10^2^ | 10^2^ | 10^4^ |
| *T_1_ phage* | 10^4^ PFU/mL | 10^2^ | 10^2^ | 10^2^ | 10^4^ |
| *T_1_ phage* | 10^5^ PFU/mL | 10^2^ | 10^2^ | 10^2^ | 10^4^ |
| *T_1_ phage* | 10^6^ PFU/mL | 10^2^ | 10^2^ | 10^2^ | 10^4^ |
| DNA sequence | 1.7ng/mL | 10^2^ | 10^2^ | 10^2^ | 10^4^ |
| DNA sequence | 6.7ng/mL | 10^2^ | 10^2^ | 10^2^ | 10^4^ |
| DNA sequence | 26.7ng/mL | 10^2^ | 10^2^ | 10^2^ | 10^4^ |

Supplementary Table 2 the CSF simulated samples for exploring linearity of spike-ins’ quantification

| Spike-ins | The concentration of spike-ins | *S. aureus* (CFU/mL) | *E. coli* (CFU/mL) | *K.pastoris* (CFU/mL) | Human cells (/mL) |
| --- | --- | --- | --- | --- | --- |
| *Thermus thermophilus* | 10^3^CFU/mL | 10^2^ | 10^2^ | 10^2^ | 10^4^ |
| *Thermus thermophilus* | 10^3^ CFU/mL | 10^3^ | 10^3^ | 10^3^ | 10^4^ |
| *Thermus thermophilus* | 10^3^ CFU/mL | 10^4^ | 10^4^ | 10^4^ | 10^4^ |
| *T_1_ phage* | 10^4^ PFU/mL | 10^2^ | 10^2^ | 10^2^ | 10^4^ |
| *T_1_ phage* | 10^4^ PFU/mL | 10^3^ | 10^3^ | 10^3^ | 10^4^ |
| *T_1_ phage* | 10^4^ PFU/mL | 10^4^ | 10^4^ | 10^4^ | 10^4^ |
| DNA sequence | 2ng/300uL | 10^2^ | 10^2^ | 10^2^ | 10^4^ |
| DNA sequence | 2ng/300uL | 10^3^ | 10^3^ | 10^3^ | 10^4^ |
| DNA sequence | 2ng/300uL | 10^4^ | 10^4^ | 10^4^ | 10^4^ |

Supplementary Table 3 the CSF simulated samples for exploring the effect of human cells on spike-ins quantification

| Spike-ins | The concentration of spike-ins | *S. aureus* (CFU/mL) | *E. coli* (CFU/mL) | *K.pastoris* (CFU/mL) | Human cells (/mL) |
| --- | --- | --- | --- | --- | --- |
| *Thermus thermophilus* | 10^3^ CFU/mL | 10^2^ | 10^2^ | 10^2^ | 10^3^ |
| *Thermus thermophilus* | 10^3^ CFU/mL | 10^2^ | 10^2^ | 10^2^ | 10^4^ |
| *Thermus thermophilus* | 10^3^ CFU/mL | 10^2^ | 10^2^ | 10^2^ | 10^5^ |
| *Thermus thermophilus* | 10^3^ CFU/mL | 10^2^ | 10^2^ | 10^2^ | 10^6^ |
| *T_1_ phage* | 10^4^ PFU/mL | 10^2^ | 10^2^ | 10^2^ | 10^3^ |
| *T_1_ phage* | 10^4^ PFU/mL | 10^2^ | 10^2^ | 10^2^ | 10^4^ |
| *T_1_ phage* | 10^4^ PFU/mL | 10^2^ | 10^2^ | 10^2^ | 10^5^ |
| *T_1_ phage* | 10^4^ PFU/mL | 10^2^ | 10^2^ | 10^2^ | 10^6^ |
| DNA sequence | 2ng/300uL | 10^2^ | 10^2^ | 10^2^ | 10^3^ |
| DNA sequence | 2ng/300uL | 10^2^ | 10^2^ | 10^2^ | 10^4^ |
| DNA sequence | 2ng/300uL | 10^2^ | 10^2^ | 10^2^ | 10^5^ |
| DNA sequence | 2ng/300uL | 10^2^ | 10^2^ | 10^2^ | 10^6^ |

Supplementary figure 1: The concentration of host DNA. The blue dots present the host reads numbers and the black solid lines show the tendency of host reads numbers on gradients T1 phage solution arranging 10^3^ PFU/mL to 10^6^ PFU/mL


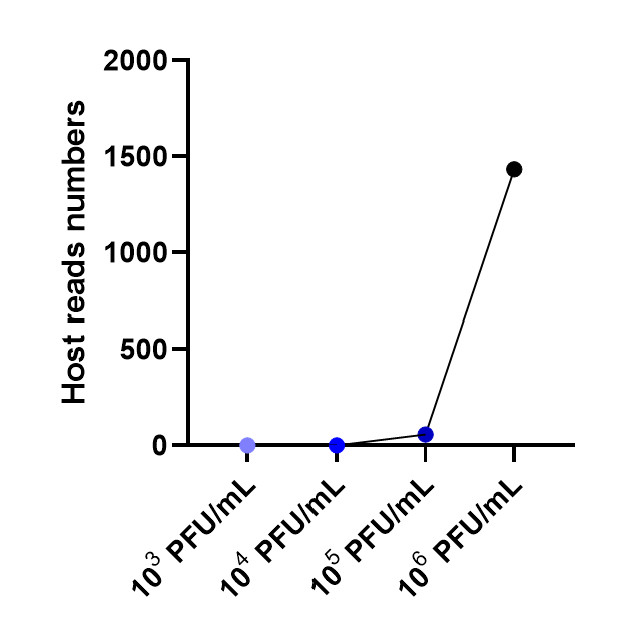


Supplementary figure 2: the effects of human cell concentrations on pathogen (a) and spike-ins(b) reads number. The red, green and blue dots on left picture present the reads number of pathogens *E.coli*, *S.aureus* and *Komagataella pastoris* on simulated samples, while on the right, the red, green and blue ones present the *Thermus thermophilus*, *T_1_ phage* and DNA sequence reads number. The solid lines on both picture reflects the tendency with the human cells concentration increasing.


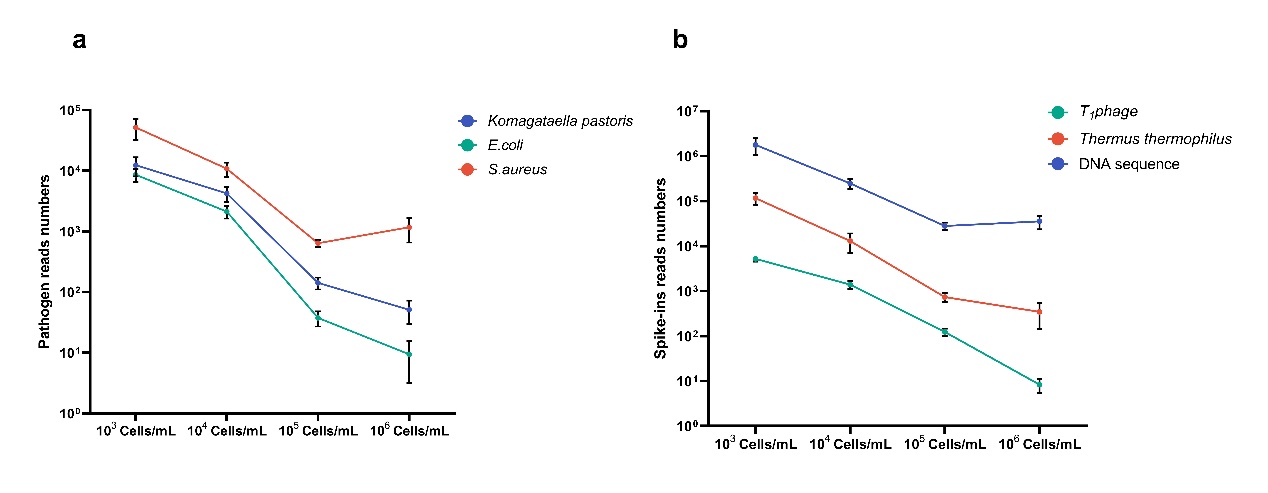

Supplement: Supplemental file 1 — Supplemental methods, Tables S1 to S3, and Fig. S1 and S2 [file spectrum.01139-23-s0001.docx]
